# P4HB regulates tumor-associated macrophage polarization and chemotaxis by enhancing IL-6 cytokine secretion in glioblastoma

**DOI:** 10.1097/MS9.0000000000004049

**Published:** 2025-10-09

**Authors:** Minkai Wang, Zeyu Ma, Xiaotao Wang, Zilong Wang, Wenqing Wang, Zhenyu Zhang, Ran Li, Xianzhi Liu, Dongling Pei

**Affiliations:** a Department of Neurosurgery, The First Affiliated Hospital of Zhengzhou University, Zhengzhou, Henan, China; b School of Medicine, Hangzhou City University, Hangzhou, Zhejiang, China; c Institute of Infection and Immunity, Henan Academy of Innovations in Medical Science, Zhengzhou, Henan, China

**Keywords:** glioma, IL-6/STAT3, microglia polarization, P4HB, tumor microenvironment

## Abstract

**Background:**

Prolyl 4-hydroxylase subunit beta (P4HB) has been linked to glioma progression and treatment resistance; however, its role in the tumor microenvironment regulation remains unclear.

**Materials and Methods:**

A total of 90 human glioma samples and 30 normal traumatic brain injury (TBI) tissues from patients undergoing surgical resection were collected from the First Affiliated Hospital of Zhengzhou University for immunohistochemical (IHC) staining. The signaling pathway mechanism regulating prognosis and tumor proliferation was explored through bioinformatics analysis and functional assays via *P4HB* knockdown.

**Results:**

The IHC analysis of 90 glioma and 30 TBI tissues revealed that P4HB expression correlated with tumor malignancy, especially the WHO grade 4 and grade 2 (*P* < 0.0001). Furthermore, transcriptomic data analysis identified P4HB as a prognostic marker associated with poor survival and tumor-associated macrophage infiltration (*P* < 0.05 in all cohorts). Using shRNA lentiviral constructs, stable *P4HB* knockdown glioblastoma (GBM) cell lines were generated. mRNA sequencing revealed significant downregulation of the interleukin (IL)-6/signal transducer and activator of transcription 3 (IL-6/STAT3) signaling axis. Functional studies showed that P4HB deficiency resulted in reduced IL-6 secretion (*P* < 0.0001), suppressed M2 polarization of tumor-associated microglia, and inhibited glioma cell growth both *in vitro* and *in vivo*, with IL-6 neutralization recapitulating these effects. Mechanistically, P4HB promoted STAT3 phosphorylation in microglia, driving their pro-tumorigenic M2 phenotype.

**Conclusion:**

These findings establish P4HB as a regulator of glioma progression via IL-6/STAT3-mediated microglial polarization, highlighting its potential as a therapeutic target for GBM.

## Introduction

The tumor microenvironment (TME) of gliomas consists not only of neoplastic cells but also diverse stromal and immune cell populations, with tumor-associated macrophages (TAMs) representing the dominant immune infiltrate in diffuse gliomas^[1]^. Macrophages exhibit two distinct activation states-classically activated (M1) and alternatively activated (M2) phenotypes. Emerging evidence highlights TAMs as critical mediators of the tumor-immune cross talk, fostering immunosuppressive microenvironments that accelerate glioma progression^[2,3]^.

**HIGHLIGHTS**

- Prolyl 4-hydroxylase subunit beta (P4HB) drives glioblastoma progression by enhancing interleukin (IL)-6 secretion, which promotes STAT3-mediated M2 polarization of tumor-associated microglia, fostering an immunosuppressive tumor microenvironment.
- High P4HB expression correlates with poor glioma patient prognosis and increased macrophage infiltration, particularly pro-tumorigenic M2-like phenotypes.
- Targeting P4HB suppresses IL-6/STAT3 signaling, reducing microglial chemotaxis and M2 polarization, thereby inhibiting tumor growth *in vitro* and *in vivo*.

During early tumorigenesis, TAMs typically adopt an M1 phenotype characterized by high expression of CD86, major histocompatibility complex II (MHC-II), and IL-12, while expressing low levels of CD206, CD163, arginase-1 (Arg-1), and IL-10. M1 macrophages exert antitumor effects through enhanced phagocytosis, cytotoxicity, and antigen presentation^[4]^. Conversely, in advanced disease stages, TAMs polarize toward an M2 phenotype marked by reduced CD86/MHC-II/IL-12 expression and elevated CD206/CD163/Arg-1/IL-10 expression, promoting tumor angiogenesis, immunosuppression, and metastasis^[5–7]^.

Glioma TAMs are primarily derived from either peripherally recruited monocytes (monocyte-derived TAMs) or resident brain microglia (microglia-derived TAMs [Mg-TAMs]). Dysregulated polarization and chemotaxis of Mg-TAMs pose major therapeutic challenges in glioma treatment^[8]^.

Cancer cells exhibit elevated protein synthesis rates, imposing substantial demand on the endoplasmic reticulum (ER) protein-folding machinery^[9]^. To mitigate ER stress – a state of imbalance between unfolded protein load and folding capacity – cells activate the unfolded protein response (UPR), a signaling network that restores ER homeostasis by augmenting folding, degradation, and transport pathways^[10]^. Central to this adaptation is the upregulation of protein disulfide isomerase (PDI). Moreover, prolyl 4-hydroxylase subunit beta (P4HB), a member of the PDI family, is a multifunctional chaperone and enzyme that catalyzes disulfide bond formation, isomerization, and repair of misfolded proteins, thereby alleviating ER stress^[11]^. Notably, *P4HB* is among the most highly expressed genes in glioblastoma (GBM), reflecting its critical role in sustaining proteostasis in these aggressive tumors^[12]^.

The present study showed that P4HB expression in glioma tissues positively correlated with tumor malignancy and poor patient outcomes, while bioinformatics analysis revealed a link between P4HB upregulation and immunosuppressive macrophage infiltration, particularly Mg-TAM M2 polarization. Functional experiments demonstrated that *P4HB* knockdown in GBM cells suppressed IL-6 secretion through STAT3 inactivation, reducing microglial chemotaxis and M2 phenotypic markers (CD206, CD163) both in coculture systems and *in vivo*. Moreover, inhibition of P4HB expression or antagonism of IL-6 secretion could both suppress the proliferation and growth of GBM in mice.

Mechanistically, P4HB modulated the IL-6/STAT3 axis to drive microglial polarization, creating an immunosuppressive niche that promoted glioma progression. These findings establish P4HB as a novel regulator of the glioma-immune cross talk and highlight its therapeutic potential for targeting tumor-associated microglia in GBM.

## Materials and methods

### Clinical samples

This study was conducted in accordance with ethical guidelines and approved by the Institutional Review Board (IRB) of the First Affiliated Hospital of Zhengzhou University (FAHZZU; Ethical Approval No. 2019-KY-176). The 90 human glioma samples and 30 traumatic brain injury (TBI) tissues from patients undergoing surgical resection were collected from the FAHZZU for immunohistochemical (IHC) staining. Informed consent was obtained from the patients, and all procedures were performed in accordance with the relevant guidelines and regulations of the FAHZZU.

### Collection and processing of GBM data from public database and FAHZZU cohort

In this study, we collect glioma sequencing data from public databases: the Cancer Genome Atlas (TCGA, https://www.cancer.gov/ccg/research/genome-sequencing/tcga) and the Chinese Glioma Genome Atlas (CGGA; http://www.cgga.org.cn/) including CGGA array (GPL4133) and CGGA RNA-seq (Illumina HisSeq). Additional analyses were performed based on our existing mRNA and proteomic datasets^[13]^. Our own raw RNA-seq data generated in this study have been deposited in the Genome Sequence Archive database under accession code HRA006184. The raw MS data-based proteomics supporting the findings of this study have been deposited in the iProX database under accession code PXD062023.

### Cell culture and reagents

The human GBM cell line T98G was obtained from the Cell Resource Center of Science (Shanghai, China). The human microglial cell line HMC3 and murine GBM cell line GL261 were sourced from American Type Culture Collection of original provider, with authentication confirmed by short tandem repeat profiling (for HMC3) and species-specific polymerase chain reaction (PCR) (for GL261) prior to use. All cell lines were tested for mycoplasma contamination and maintained for ≤10 passages post-thawing. The T98G and GL261 cells were grown in Dulbecco’s modified Eagle’s medium supplemented with 10% fetal bovine serum (FBS) (Cat No. FSP500, ExCell Bio), 100 IU/mL penicillin, and 100μg/mL streptomycin sulfate. Human microglial HMC3 cells were treated with 100ng/mL phorbol 12-myristate 13-acetate (PMA) for 48h for macrophage differentiation. HMC3 cells were cultured in MEM containing nonessential amino acids, 10% FBS, and antibiotic (100μg/mL penicillin/streptomycin). All the cells were incubated in a humidified atmosphere at 37°C in 5% CO_2_ and were free of mycoplasma contamination.

### Lentivirus transduction

Lentiviruses were produced by transfecting HEK293T cells with the pLKO.1 (for P4HB shRNA knockdown) plasmids and helper plasmids pCMV-VSVG, pMDLg-RRE (gag/pol), and pRSV-REV. The cell supernatants were harvested 48h after transfection and used to infect cells or stored at −80°C. To obtain stable cell lines, T98G and GL261 cells were infected, at a low confluence (20%), for 18h with lentiviral supernatants diluted 1:1 in normal culture medium supplemented with 5ng/mL polybrene (Sigma). After 48h of infection, the cells were subjected to puromycin selection for 1week and then passaged before use. Puromycin (1µg/mL) maintained selection pressure on stably transfected GBM cells. Lentiviral shRNAs were identified and tested, and the most effective shRNA was used. The following P4HB shRNA were used:

TRCN0000049194 (sh-hP4HB #1): 5′-GTGTGGTCACTGCAAACAGTT-3′;

TRCN0000296675 (sh-hP4HB #2): 5′-AGGTGAAATCAAGACTCACAT-3′;

TRCN0000111865 (sh-mP4HB #1): 5’-GCATTTCATCTGTGAGGCATT-3’;

TRCN0000111869 (sh-mP4HB #2): 5’-CAGCGCATACTTGAGTTCTTT-3’.

### RNA isolation and quantitative reverse transcriptase-PCR

The NucleoSpin RNA II kit (BIOKE, Netherlands) was used to extract RNA from total cells, and cDNA reverse transcription was performed using the RevertAid First Strand cDNA Synthesis Kit (Fermentas). Reverse transcriptase-PCR (RT-qPCR) was conducted with SYBR Green (Applied Bioscience) using a StepOnePlus real-time PCR system (Applied Bioscience 7500). Each sample was analyzed at least in triplicate, and glyceraldehyde-3-phosphate dehydrogenase (GAPDH) was used as an internal standard. The primers for RT-qPCR are listed as follows:

hP4HB (Forward): 5′-TCACCAAGGAGAACCTACTGGA-3′

hP4HB (Reverse): 5′-GGCAAGAACAGCAGGATGTGAG-3′

hIL-6 (Forward): 5′-ACTCACCTCTTCAGAACGAATTG-3′

hIL-6 (Reverse): 5′-CCATCTTTGGAAGGTTCAGGTTG-3′

hCD206 (Forward): 5′-GGGTTGCTATCACTCTCTATGC-3′

hCD206 (Reverse): 5′-TTTCTTGTCTGTTGCCGTAGTT-3′

hCD163 (Forward): 5′-TTTGTCAACTTGAGTCCCTTCAC-3′

hCD163 (Reverse): 5′-TCCCGCTACACTTGTTTTCAC-3′

hGAPDH (Forward): 5′-TGATGACATCAAGAAGGTGGTGAAG-3′

hGAPDH (Reverse): 5′-TCCTTGGAGGCCATGTGGGCCAT-3′

mP4HB (Forward): 5′-GTCAACTGGCTGAAGAAACGC-3′

mP4HB (Reverse): 5′-CGCTTGAGTCCACCAAGGAC-3′

mGAPDH (Forward): 5′-CATGGCCTTCCGTGTTCCTA-3′

mGAPDH (Reverse): 5′-CCTGCTTCACCACCTTCTTG-3′

### CCK-8 assay

Cells were plated in 96-well plates at a density of 5×10^3^ cells/well in 200µL of complete media or culturing medium (CM) from HMC3. The Cell Counting Kit-8 (CCK-8; Biosharp, Cat#BS350A, China) was used to determine the absorbance of the medium in each well at the first to fifth day.

### Transwell chemotaxis assays

Chemotaxis assays were conducted in Transwell 24-well plates (Corning, Cat#3422, USA) equipped with 8-µm pore filters and 6.5-mm inserts. In the upper chamber, 1×10^4^ HMC3 cells were suspended in 200µL serum-free medium, while the lower chamber contained 1×10^5^ T98G cells cultured in 500µL medium supplemented with 10% FBS. Following a 24h incubation at 37°C under 5% CO_2_, the migrated cells were fixed with paraformaldehyde (15 min) and stained with 0.1% crystal violet (15 min). Nonmigrated cells on the upper filter surface were gently removed using a cotton swab, and residual dye was washed away with phosphate-buffered saline (PBS). Migrated cells adhering to the filter were quantified microscopically at 200× magnification, with each condition tested in triplicate.

### Immunoblotting

Cells were lysed with 1 mL of lysis buffer (20 mM Tris-HCl pH 7.4, 2 mM ethylenediamine tetraacetic acid (**EDTA**), 25 mM sodium fluoride (**NaF**), and 1% Triton X-100), containing protease inhibitors (Cocktail, Bimake) for 10 min at 4°C. After centrifugation at 12×10^3^*g* for 10 min, protein concentrations were measured, and equal amounts of lysate proteins were separated via sodium dodecyl sulfate–polyacrylamide gel electrophoresis (SDS-PAGE). The proteins were transferred onto a nitrocellulose membrane, which was then blocked with a blocking buffer (5% skim milk in TBS-T) for 1h at room temperature and incubated with primary antibodies. Immunoblotting (IB) was performed using specific antibodies and secondary anti-mouse or anti-rabbit antibodies conjugated to horseradish peroxidase (Amersham Biosciences). Visualization was performed through chemiluminescence. The antibodies used for IB were as follows: P4HB at 1:2000 (Cat#PTM-5314, PTM-biolab), CD86 at 1:2000 (Cat#A21198, ABclonal), MHC II at 1:1000 (Cat#bs-8481R, Bioss), CD163 at 1:2000 (Cat#PA5-78961, Invitrogen), CD206 at 1:1000 (Cat#18704-1-AP, Proteintech), p-STAT3(Y705) at 1:2000 (Cat#39595, Proteintech), STAT3 at 1:1000 (Cat#60199-1-Ig, Proteintech), programmed cell death protein ligand 1 (PD-L1) at 1:2000 (Cat#66248-1-Ig, Proteintech), and GAPDH at 1:5000 (Cat#ab8245, Abcam).

### IHC analysis

The TBI tissues and glioma tissues were post-fixed in 4% paraformaldehyde, embedded in paraffin, and sliced into 8-µm-thick coronal sections. Next, the sections were deparaffinized with xylene and rehydrated using a descending ethanol gradient, followed by antigen retrieval. The sections were then treated with 0.3% H_2_O_2_ for 20 min to inhibit endogenous peroxidase activity and incubated with 5% goat serum for 30 min. Subsequently, the sections were incubated with the indicated primary antibodies against P4HB at 1:200 (Cat#PTM-5314, PTM-biolab) for 12h at 4°C, washed with PBS, and incubated with biotinylated secondary antibodies at 37°C for 60 min. After washing with PBS, the sections were stained with diaminobenzidine and counterstained with hematoxylin.

### Subcutaneous tumor model

Mice experiments were approved by the FAHZZU Animal Welfare Committee. Six-week-old female C57BL/6 mice were purchased from the GemPharmatech Co., Ltd. (Nanjing, China), and housed in a specific pathogen-free environment for 3days before use. All the mice were acclimated under standard laboratory conditions (ventilated room, 25°C, 55–65% humidity, 12h light/dark cycle) and had free access to standard water and food. The mice were randomly allocated into two groups (*n* =6 per group). sh-NC or sh-P4HB #1 GL261 cells were subcutaneously injected into the flanks of nude mice (5×10^6^ cells per mouse). Seven days after injecting the mice with tumor cells, the tumor volume of mice was measured every 5days. Until the maximal tumor size was near 8 cm^3^, the mice were then sacrificed by cervical dislocation. The tumors were removed and their weight and volume were measured and recorded. Subsequently, tumor tissues were fixed with optimal cutting temperature compound for IHC and western blotting (WB). For IL-6 neutralization experiments *in vivo*, GL261 cells (5×10^6^ cells in 100µL of PBS) were injected into the right flank of the C57BL/6 mice. Mice were randomly allocated to experimental groups using a computer-generated randomization table (*n* =5 per group) to ensure balanced baseline tumor burden. The mice were treated with 25µg of anti-IL-6 antibody (eBioscience) or equal IgG, which was injected intraperitoneally every 4days during the course of the experiment, starting on the seventh day of tumor inoculation.

### Statistical analysis

Comparisons between groups were performed using an unpaired two-tailed Student’s *t*-test; analysis of variance (ANOVA) was used for multivariate analysis. Survival curves were plotted using the Kaplan–Meier method, and *P*-values were determined using the log-rank test. GraphPad Prism 7 (GraphPad Inc., La Jolla, CA, USA) was used for statistical analysis and graphing. The *P*-values are indicated by asterisks in the figures. *P < 0.05, **P < 0.01, ***P < 0.001, ****P < 0.0001. *P* < 0.05 was considered significant.

## Results

### High expression of P4HB in glioma tissues correlates with poor patient prognosis

**Figure 1.**
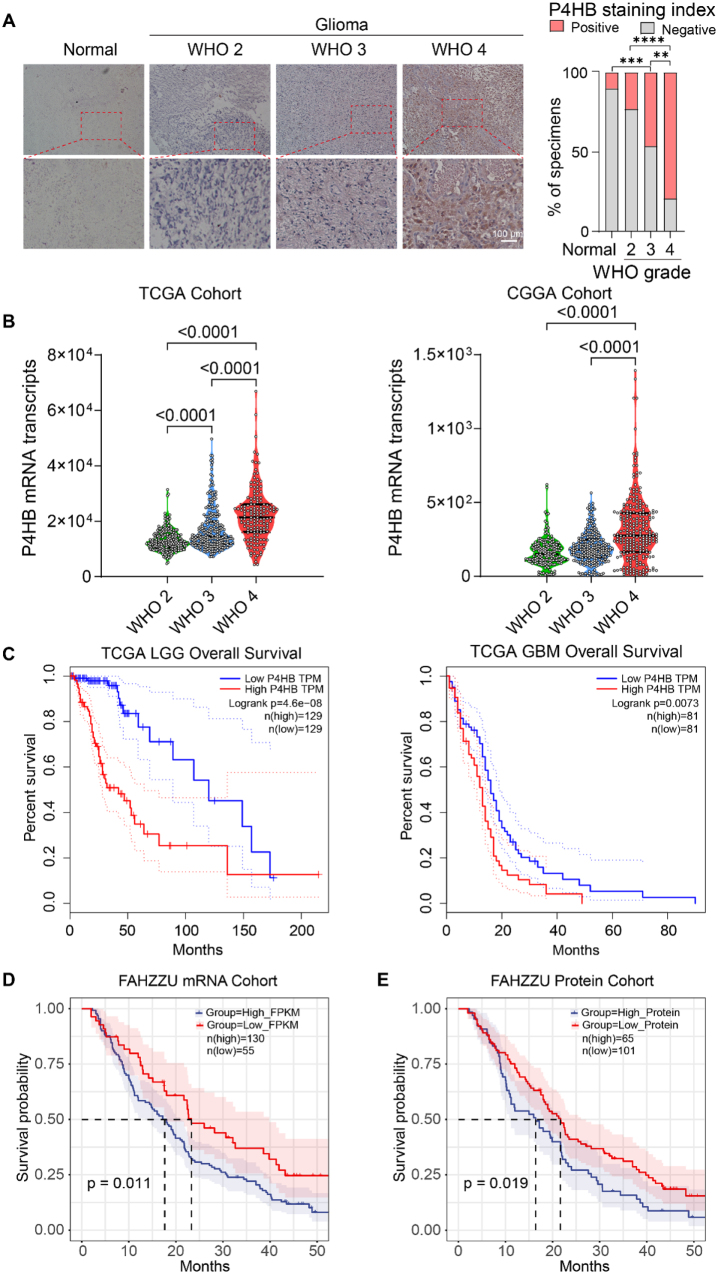
High expression of P4HB in glioma tissues correlates with poor patient prognosis. (A) Representative IHC images (10× and 40×) showing P4HB expression in different WHO grades of glioma and TBI tissues. (B) P4HB mRNA levels of differential glioma grades in TCGA/CGGA cohorts. (C) Survival analysis of TCGA LGG/GBM patients grouped by P4HB expression (high P4HB TPM vs. Low P4HB TPM). (D) Kaplan–Meier analysis of overall survival in glioma patients stratified by P4HB expression levels (high vs. low) in the FAHZZU mRNA-seq cohort. (E) Kaplan–Meier survival analysis of FAHZZU glioma patients stratified by P4HB protein expression levels (high vs. low) in our mass spectrometry/mass cytometry (MS/MC) proteomic dataset. **P < 0.01, ***P < 0.001, ****P < 0.0001. Data are presented as mean±SD (A, right, and B). Statistical analyses are performed using nonparametric one-way ANOVA (A, right, and B) and log-rank test (C–E).

To investigate the expression of P4HB in human glioma and TBI tissues, IHC staining was performed on 90 glioma tissues (WHO II–IV grades) and 30 non-matched TBI tissues from trauma patients (Fig. 1A, left panel). IHC scoring revealed that P4HB expression increased with higher WHO grades, and the scores in tumor tissues were significantly higher than those in TBI tissues (*P* < 0.05; Fig. 1A, right panel). An analysis of transcriptomic sequencing data from TCGA and the CGGA databases further showed that *P4HB* mRNA levels escalated with increasing WHO grades (Fig. 1B). Survival analysis in the TCGA cohort demonstrated that patients with glioma with high P4HB expression exhibited significantly lower overall survival than those with low P4HB expression in both the primary low-grade glioma (LGG) and GBM patients with standardized radiotherapy and chemotherapy after surgical resection (Fig. 1C). Additionally, analysis of preexisting RNA sequencing and proteomic data sourced from the primary GBM cohorts of the FAHZZU revealed that there was a negative correlation between P4HB and prognosis (Fig. 1C and 1D).

### Correlation between P4HB and immune cell infiltration in glioma tissues

**Figure 2.**
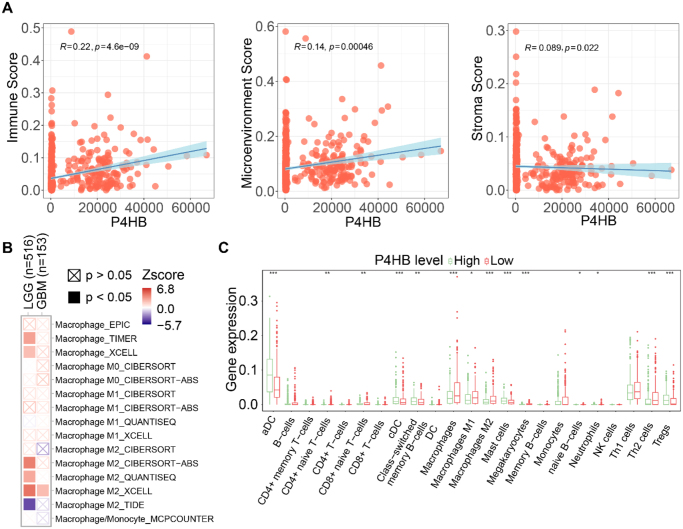
Correlation between P4HB and immune cell infiltration in glioma tissues. (A) Pearson correlation analysis of P4HB expression levels with immune scores, tumor microenvironment scores, and stromal scores using The Cancer Genome Atlas (TCGA) data. (B) The relationship between immune cell infiltration and P4HB expression in low-grade glioma (LGG) and glioblastoma (GBM) based on TCGA data. (C) Box plots of immune cell infiltration scores for high- and low-P4HB expression groups drawn based on TCGA data. *P < 0.05, **P < 0.01, ***P < 0.001. Data are presented as mean± SD (C). Statistical analyses are performed using nonparametric one-way ANOVA (B and C) and log-rank test (A).

Tumor-infiltrating immune cells, as a critical component of TME, are closely associated with cancer initiation, progression, and metastasis^[14,15]^. In this study, eight computational algorithms (EPIC, TIMER, CIBERSORT, CIBERSORT-ABS, QUANTISEQ, XCELL, MCPCOUNTER, and EPIC) were employed to investigate the potential relationship between *P4HB* expression and the infiltration levels of distinct immune cell subsets in GBM and LGG from TCGA database. Correlation analyses of P4HB expression with immune score, TME score, and stromal score revealed a strong positive association between P4HB and macrophage immune infiltration (Fig. 2A). Further subgroup analysis demonstrated that P4HB expression was specifically correlated with the M2 polarization of macrophages (Fig. 2B, 2C).

### P4HB reduces IL-6 secretion in glioma cells via the JAK/STAT3 signaling pathway

**Figure 3.**
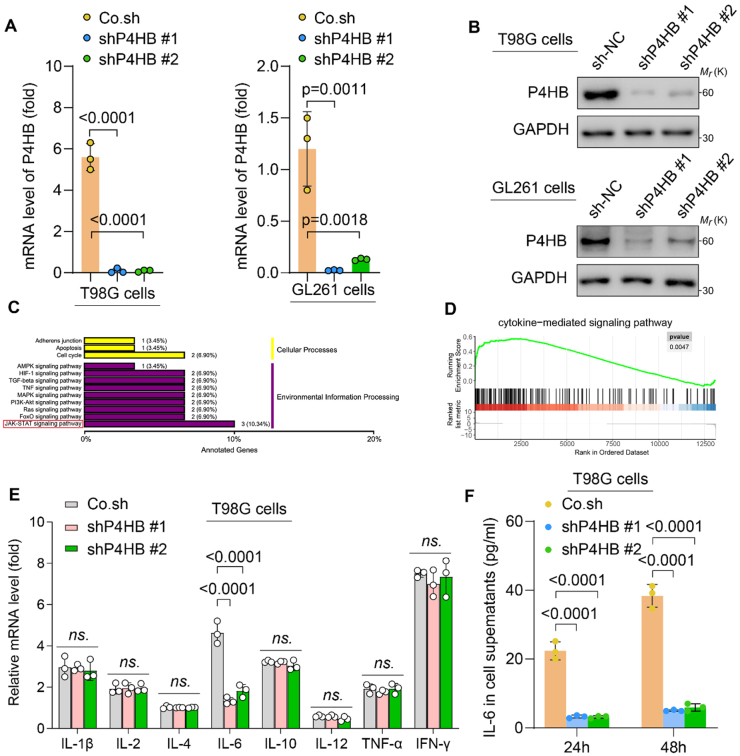
P4HB reduces IL-6 secretion in glioma cells via the JAK/STAT3 signaling pathway. (A-B) Knockdown of *P4HB* in T98G and GL261 cell lines is validated by (A) RT-qPCR (mRNA level) and (B) WB (protein level). (C) Functional categorization of downregulated genes in *P4HB* knockdown T98G cells, based on GO annotations. Detailed information is shown in Available at, Supplemental Digital Content Table S1: http://links.Lww.Com/MS9/A980. **(D)** GSEA of the JAK-STAT3 pathway to assess specific enrichment of *P4HB* knockdown versus control cells. (E) Expression of several JAK/STAT3 pathway-related cytokine genes identified via qPCR. (F) Expression of IL-6 product in the medium of sh-NC and sh-P4HB groups in T98G and GL261 cells after culturing for 24 and 48h. Data from at least three independent experiments were analyzed and are presented as mean±SD (A, B, E, and F). Statistical analyses are performed using the nonparametric one-way ANOVA (A, B, E, and F) and log-rank test (C and D).

In human GBM T98G and murine GBM GL261 cell lines, RT-qPCR (Fig. 3A) and WB (Fig. 3B) analyses showed that the mRNA and protein expression levels of P4HB were significantly reduced in sh-P4HB #1 and sh-P4HB #2 cells compared with those in sh-NC cells. Transcriptomic sequencing of sh-NC and sh-P4HB #1 T98G cells, followed by gene ontology (GO) enrichment analysis, revealed that *P4HB* knockdown significantly suppressed the JAK/STAT3 signaling pathway, as shown in Fig. 3C. Gene set enrichment analysis (GSEA) further validated that P4HB played a key regulatory role in cytokine-mediated signaling pathways (Fig. 3D). The JAK/STAT3 pathway is known to upregulate the levels of multiple proinflammatory cytokines, which are critical for TME modulation^[16–18]^. To further investigate the role of P4HB in cytokine regulation through the JAK/STAT3 pathway, we quantified the mRNA expression levels of multiple JAK/STAT3-associated cytokines in T98G cells following *P4HB* knockdown. We found that the changes in P4HB expression only influenced IL-6 expression (3.293±0.335, *P* < 0.0001; 2.797±0.244, *P* < 0.0001; Fig. 3E); they had little effect on the expression of IL-1β, IL-2, IL-4, IL-10, IL-12, tumor necrosis factor alpha, and interferon gamma in T98G cells. Enzyme-linked immunosorbent assay results confirmed that *P4HB* knockdown significantly reduced IL-6 secretion in the culture medium (Fig. 3F).

### Downregulation of P4HB in GBM cell lines inhibits chemotaxis and the M2 polarization of cocultured microglia

**Figure 4.**
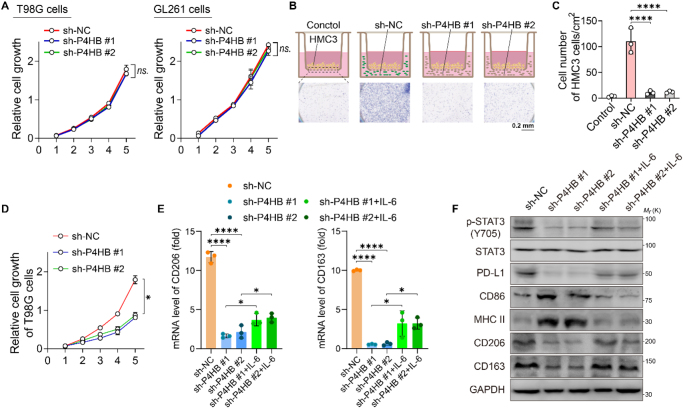
Downregulation of P4HB in T98G inhibits chemotaxis and M2 polarization of cocultured microglia. (A) Effect of *P4HB* knockdown on tumor cell growth in T98G and GL261 cells. (B) Coculture of PMA-treated microglia with differently treated glioblastoma cells. (C) Quantification of microglial chemotaxis toward T98G cells after coculture as shown in (B). (D) Effect of *P4HB*knockdown on glioblastoma cell growth when cocultured with activated microglia (as in B). (E) Microglia markers identified via RT-qPCR after culturing with *P4HB* knockdown medium or IL-6 added medium (100pg/ml). (F) Protein expression levels of microglial polarization markers, PD-L1 and shown in (E). *P < 0.05, ****P < 0.0001. Data from at least three independent experiments were analyzed and are presented as mean±SD (A, C, D, and F). Statistical analyses were performed using the nonparametric one-way ANOVA (A, C, D, and F).

**Figure 5.**
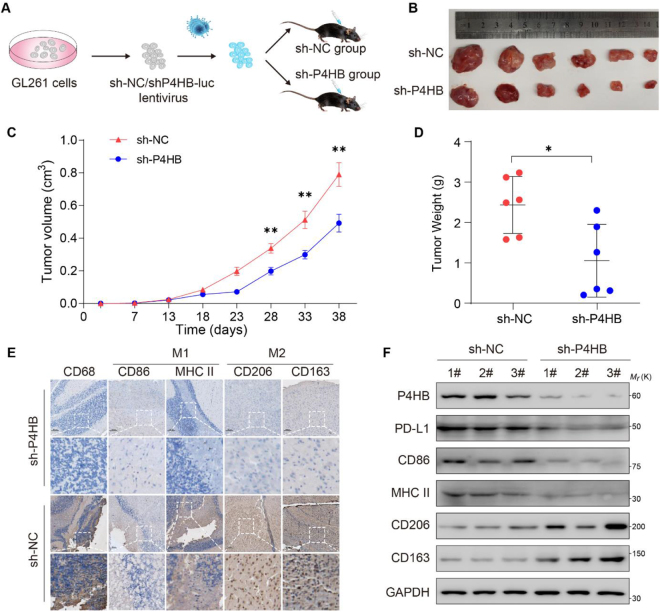
P4HB promotes tumor progression via M2 microglial polarization in a subcutaneous tumor model. (A) Schematic of the experimental design for the GL261 subcutaneous tumor model. (B) Representative image of tumors from each group. (*N* =6 per group). (C) Effect of *P4HB* knockdown on the growth of GL261 xenograft tumors. Tumor volume was measured every 5days from the seventh day after tumor implantation. (D) Tumor weight was measured at the end of the study. (E) Immunohistochemical staining of macrophages (CD68^+^), M2 (CD206^+^ and CD163^+^) macrophages, and M1 (CD86^+^ and MHC II^+^) macrophages in tumor sections. Scale bar: 100μm. (F) Representative tumor tissues from each group were prepared and subjected to western blotting analysis. **P < 0.01. Statistical analyses were presented as mean±SD and performed using a two-tailed Student’s *t*-test (C and D).

Knockdown of *P4HB* did not significantly affect the proliferation of GBM cell lines (Fig. 4A). Considering our previous findings that microglia with macrophage-like characteristics are involved in the regulatory mechanism underlying the poor prognosis of patients with high P4HB expression, human HMC3 microglia were induced by PMA to differentiate into macrophage-like cells via adherent culture. The chemotactic ability of microglia was assessed by coculturing macrophages with different groups of GBM cells in transwell chambers (Fig. 4B). Chemotaxis assay results showed that the number of chemotactic macrophages in the T98G-sh-P4HB group was significantly lower than that in the T98G-sh-NC group (Fig. 4C). The CCK-8 assay was used to detect the growth of differentially treated T98G cells, cultured with supernatant culture medium of HMC3 cells (Fig. 4D). The proliferation of T98G cells of *P4HB* knockdown was markedly impaired upon microglia medium (Fig. 4D), whereas the GBM cells cultured alone did not exhibit growth inhibition (Fig. 4A), which suggested that the macrophage-tumor cell cross talk drives this phenotypic change. RT-qPCR and WB analyses revealed that CM of sh-P4HB T98G cells significantly reduced the protein expression of CD206 and CD163 (M2 polarization markers) on the surface of HMC3 microglia (Fig. 4E, 4F). Furthermore, the expression of PD-L1 in shP4HB-CM-educated macrophages was (Fig. 5F). Conversely, IL-6 stimulation increased the expression of CD206, CD163, PD-L1, and STAT3 phosphorylation; CD86 and MHC-II (M1-related markers) expression increased before IL-6 treatment but decreased after stimulation (Fig. 4E). The RT-qPCR results corroborated these findings at the transcriptional level (Fig. 4F).

### P4HB enhances tumor promotion via M2 microglial polarization in a subcutaneous tumor model

To validate the aforementioned findings *in vitro*, we generated orthotopic xenografts in C57BL/6 mice with normal immunity using *P4HB* knockdown GL261 cells (Fig. 5A). Compared to those in the control xenograft mice, the tumor volumes showed a statistically significant decrease in P4HB-deficient mice from 28th day after tumor implantation (Fig. 5B). Moreover, subcutaneous tumors harvested at the end of the experiment weighed significantly less in P4HB-deficient mice than in their control counterparts (Fig. 5C, 5D). IHC and WB analyses revealed that compared to the sh-P4HB group, the sh-NC control group exhibited increased macrophage recruitment in TME (Fig. 5E, 5F). Furthermore, the proportion of polarized M2 (CD206^+^ and CD163^+^) macrophages surpassed that of M1 (CD86^+^ and MHC II^+^) macrophages (Fig. 5E, 5F).

### A neutralizing antibody, blocking IL-6 of P4HB downstream, inhibits tumor growth

**Figure 6.**
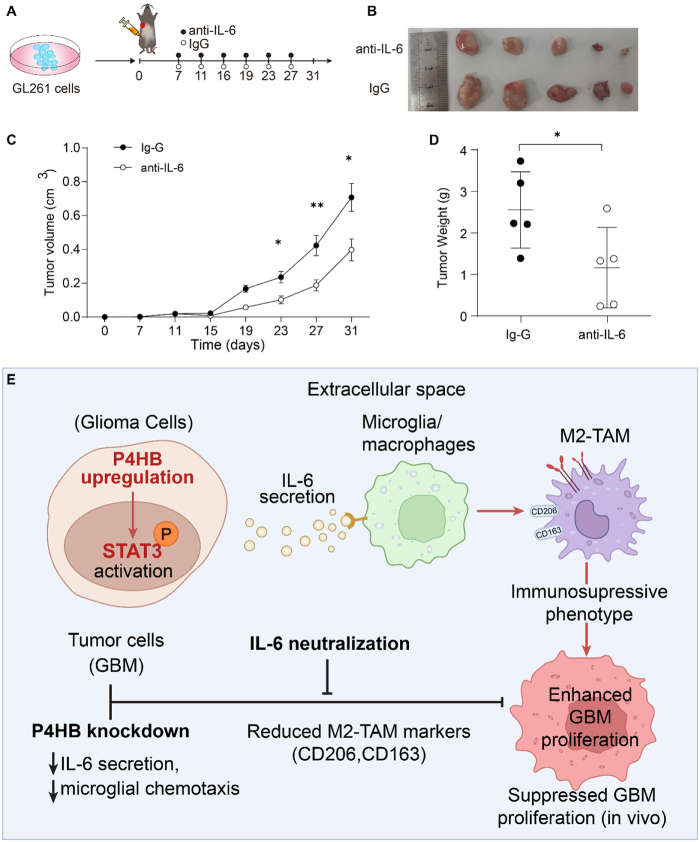
A neutralizing antibody, blocking IL-6 of P4HB downstream, inhibits tumor growth. (A) C57BL/6 mice were implanted with GL261 glioblastoma cells and administered intraperitoneally either a neutralizing anti-IL-6 monoclonal antibody or a control IgG at a dose of 25µg per mouse on day 7, 11, 16, 19, 23 and 27 (*n* =5 per group). (B) Representative image of tumors from each group. (*N* =5 per group). (C) The tumor volumes were monitored and recorded every 4 days from the seventh day. (D) Tumors excised on day 31 were weighed. (E) Schematic diagrams presented the mechanisms of P4HB in regulating macrophages and thus enhancing GBM progression. *P < 0.05, **P < 0.01. Statistical analyses were presented as mean±SD and performed using a two-tailed Student’s *t*-test (C and D).

P4HB was found to activate the IL-6-STAT3 signaling pathway and played a critical role in modulating IL-6 levels within TME. To investigate the contribution of IL-6 to tumor progression *in vivo*, GL261 cells were implanted into mice, followed by intraperitoneal administration of either a neutralizing anti-IL-6 monoclonal antibody or a control rat IgG (Fig. 6A). Treatment with the IL-6 neutralizing antibody resulted in a significant suppression of tumor growth, as demonstrated in Fig. 6B-D. Taken together, these findings demonstrated that the high expression of *P4HB* in GBM cells derived the resection of IL-6 and GBM-derived IL-6 could increase the M2 polarization phenotype by activating STAT3 signaling, and promoted macrophage PD-L1 expression, thereby contributing to GBM progression (Fig. 6E).

## Discussion

This study demonstrates that P4HB plays a critical role in GBM progression by regulating TAM polarization and chemotaxis through IL-6/STAT3 signaling to enhance IL-6 secretion. The IHC analysis of 90 glioma tissues revealed a striking correlation between P4HB expression and tumor malignancy grade, consistent with pan-cancer analysis showing P4HB overexpression in multiple tumor types^[19,20]^. Notably, our transcriptomic data from public cohorts and our institutional cohort demonstrate that high P4HB expression is an indicator of poor survival in both patients with LGG and GBM, aligning with the findings of Sun *et al* that P4HB serves as a prognostic marker in malignant gliomas^[20,21]^. P4HB has been shown to negatively correlate with various immune cells in TME, including T cells, B cells, and macrophages, suggesting its role as a pro-oncogene with immunosuppressive, pro-angiogenic, and anti-inflammatory effects^[22]^. T cells can internalize P4HB, which benefits their activation, proliferation, adhesion, and migration^[23]^. Previous study reported that regulatory T cells can induce the activation of IL-6–STAT3 signaling to facilitate the stemness of glioma cells^[24]^. In gliomas, high P4HB levels were linked to activated STAT3 signaling and increased IL-6 secretion, driving microglia toward a pro-tumorigenic M2 phenotype (CD206^+^/CD163^+^). This aligns with prior reports in breast and ovarian cancer, where P4HB was found to promote immune evasion through distinct pathways such as collagen processing and chemoresistance^[25,26]^, underscoring its conserved role in TME remodeling. Consistent with the findings in gastric cancer, where P4HB overexpression was reported to correlate with a poor prognosis and hypoxia-associated tumor progression, our study demonstrated the critical role of P4HB in GBM TME regulation through macrophage polarization^[27]^.

Notably, low P4HB expression triggers the TGF signaling pathway; inhibiting this pathway boosts immune responses in the TME, leading to M1-type macrophage polarization, which induces the Fenton response and subsequent tumor cell ferroptosis, offering a potential approach for cancer therapy^[28]^. Our findings revealed its additional role in shaping the immunosuppressive GBM microenvironment through macrophage reprogramming. The functional studies in our research revealed that *P4HB* knockdown significantly reduced IL-6 secretion, thereby suppressing the M2 polarization of microglia and inhibiting glioma cell growth both *in vitro* and *in vivo*. This is consistent with the known role of IL-6 in promoting TAM accumulation and immunosuppression across cancers^[2]^. In GBM, TAMs – especially microglia-derived subsets – are critical for tumor angiogenesis and immune suppression^[1]^. The discovery that P4HB regulates IL-6 secretion through STAT3 phosphorylation represents a major advancement in understanding the glioma-immune cross talk. While previous studies have established the role of P4HB in ER stress responses^[29]^, our study demonstrated its novel function in cytokine signaling. The selective effect on IL-6 among the multiple cytokines tested suggests that P4HB may orchestrate specific aspects of tumor-associated inflammation. This finding complements recent reports that P4HB interacts with PHGDH to regulate apoptosis pathways in esophageal cancer^[30]^ and modulates EMT in hepatocellular carcinoma^[31]^. Our *in vitro* and *in vivo* experiments showed that *P4HB* knockdown reduced microglial chemotaxis; additionally, M2 polarization provided direct evidence for the role of *P4HB* in immune cell recruitment, a phenomenon previously suggested by bioinformatic analyses^[20]^.

The role of P4HB in GBM aligns with its functions in other malignancies, such as bladder, breast, and liver cancers, wherein it regulates ER stress, metabolism, and drug resistance^[31–33]^. The therapeutic implications of targeting P4HB in glioma are notable. Our demonstration that IL-6 neutralization recapitulates the *P4HB* knockdown effects suggests potential combination strategies with existing IL-6/STAT3 inhibitors. Notably, the observed reduction in the number of CD206+/CD163+ microglia following P4HB inhibition mirrors the findings in bladder cancer, wherein P4HB suppression enhanced chemotherapy sensitivity^[33]^. The observed *in vivo* tumor growth inhibition supports the results of Sun *et al* showing the role of P4HB intemozolomide (TMZ) resistance^[21]^. This dual mechanism – targeting both tumor cell intrinsic pathways and TME immunosuppression – positions P4HB as a promising therapeutic target. Moreover, the predicted interactions between P4HB and ER-resident proteins, such as PDIA4/PDIA6, suggest the involvement of shared mechanisms in protein folding and redox regulation, which may influence TAM metabolic reprogramming^[32,34]^. For example, PDIA4 activates AKT signaling to drive chemotherapy resistance in prostate cancer, raising the possibility of convergent pathways in GBM^[35]^. Collectively, our results reveal the critical role of P4HB^+^ GBM cells in regulating tumor immune evasion, suggesting that anti-P4HB treatment may effectively improve the efficacy of anti-PD-1 therapy. These findings provide a new perspective for understanding the role of TAMs in regulating antitumor immunity and new directions for developing effective immunotherapeutic strategies for GBM.

The tumor-intrinsic mechanism centers on P4HB’s canonical function as a PDI family member: mitigating ER stress to sustain proteostasis in GBM cells. GBM cells exhibit exceptionally high rates of protein synthesis due to oncogenic signaling (e.g., EGFR amplification) and nutrient stress, which overwhelm the ER’s folding capacity and trigger ER stress^[9,10]^. P4HB alleviates this stress by catalyzing disulfide bond formation and repairing misfolded proteins, thereby preventing ER stress-induced apoptosis^[11]^. Notably, our *in vitro* CCK-8 assays showed *P4HB* knockdown did not reduce GBM cell proliferation in monoculture – suggesting the intrinsic role of P4HB is not to drive proliferation, but to promote cell survival under stressful conditions (e.g., hypoxia, nutrient deprivation) that are hallmark of the GBM microenvironment. This survival advantage is foundational: without P4HB, GBM cells would be more susceptible to ER stress-induced death, even in the absence of immune modulation.

While this study established P4HB as a regulator of IL-6/STAT3-mediated TAM polarization, several questions remain. First, We used TBI tissues as non-neoplastic controls, as several glioma study have indicated^[36,37]^. We acknowledge that TBI-induced neuroinflammation may alter P4HB expression due to reactive gliosis, ischemia, or immune cell infiltration. Prior studies^[29]^ report that TBI triggers transient upregulation of ER stress markers (including P4HB) and inflammatory cytokines (e.g., IL-6), potentially confounding baseline comparisons with glioma tissues. However, our use of non-matched TBI samples aimed to minimize acute-phase inflammatory bias by selecting tissues from chronic-phase resection specimens without active necrosis or infection. Second, the direct impact of P4HB on microglia versus tumor cells requires further clarification, as paracrine signaling from GBM cells likely drives TAM polarization^[38]^. Third, the role of P4HB in the ER stress-TAM cross talk, given its function as an ER chaperone^[29]^, merits further investigation. Additionally, translating P4HB inhibitors to the clinic will require addressing blood–brain barrier (BBB) permeability and off-target effects, as P4HB is essential for normal protein folding. Future studies should explore these avenues while advancing P4HB-targeted agents toward clinical translation for glioma patients. Translational studies are also needed to validate P4HB as a biomarker for TAM infiltration and patient prognosis in larger glioma cohorts.

Clinically, this dual mechanism underscores the advantage of targeting P4HB over single-pathway interventions. For instance, inhibiting IL-6 alone would block TAM polarization but leave intact P4HB’s intrinsic role in GBM cell survival – allowing residual tumor cells to persist and adapt. Conversely, targeting ER stress (e.g., with PDI inhibitors) would compromise tumor cell fitness but not address pre-existing immunosuppression. Only by targeting P4HB can both mechanisms be disrupted simultaneously: reducing GBM cell survival under stress and reversing TAM-mediated immunosuppression. This duality also explains why P4HB expression correlates with poor prognosis across glioma grades: in LGG, where TAM infiltration is lower, P4HB’s intrinsic survival function may drive early progression; in GBM, where TAMs dominate the TME, the immune-modulatory mechanism becomes increasingly critical. Future preclinical studies should test this hypothesis by evaluating P4HB inhibitors and targeting IL-6 drugs in both LGG and GBM models, and by stratifying efficacy based on TAM infiltration levels. In addition, future research would focus on developing better delivery materials or mediums to assist drugs in passing through BBB. Ultimately, understanding P4HB’s dual mechanism will be essential to developing therapies that effectively target both the tumor and its microenvironment in GBM.

## Conclusion

In summary, by establishing P4HB as a regulator of IL-6-mediated TAM polarization in GBM, our study provides a mechanistic framework for targeting P4HB to disrupt immunosuppressive TME. This finding aligns with emerging strategies to repurpose P4HB inhibitors, which are being explored in other cancers, for GBM treatment. Future studies should prioritize developing P4HB-targeted therapies, in combination with immune checkpoint blockade or TMZ, to enhance therapeutic efficacy, leveraging the dual role of P4HB in tumor cell survival and TME modulation.

## Data Availability

The raw RNA-seq data of our cohort generated in this study have been deposited in the Genome Sequence Archive (GSA) database under accession code HRA006184 (https://ngdc.cncb.ac.cn/gsa-human/browse/HRA006184). The raw MS data-based proteomics supporting the findings of this study have been deposited in the iProX database under accession code PXD062023.The datasets that support the findings of this study are available from the corresponding author upon reasonable request.
